# Barbiturate Derivatives for Stable Aqueous Nanoscale Pigment Yellow 150 Dispersions

**DOI:** 10.3390/ma19194059

**Published:** 2026-09-23

**Authors:** Yanxiang Wu, Shaohai Fu, Changsen Du, Dong Liang, Jiacheng Zhi, Yu Guan

**Affiliations:** 1 Key Laboratory of Sustainable Low-Carbon Technologies for Textile Dyeing and Finishing, Ministry of Education, College of Textile Science and Engineering, Jiangnan University, Wuxi 214122, China; 7213007026@stu.jiangnan.edu.cn (Y.W.); shaohaifu@hotmail.com (S.F.); 1090224214@stu.jiangnan.edu.cn (J.Z.); 2 Suzhou Sunmun Technology Co., Ltd., Suzhou 215337, China; changsen.du@smcolor.com.cn (C.D.); dong.liang@smcolor.com.cn (D.L.)

**Keywords:** pigment yellow 150, barbiturate derivative, dispersant, nano-dispersion, powder wettability

## Abstract

Three structurally related barbiturate-derived dispersants were synthesized and evaluated to investigate how molecular architecture relates to the aqueous dispersion behavior of Pigment Yellow 150. BA-A and BA-B contain arylazo/hydrazone-type frameworks bearing nominal sulfonate and carboxylate termini, respectively, whereas BA-C contains an arylidene framework bearing a nominal carboxylate terminus. Their acid-form precursors were characterized by proton nuclear magnetic resonance spectroscopy, Fourier-transform infrared spectroscopy, and ultra-performance liquid chromatography–mass spectrometry. BA-A and BA-B produced Pigment Yellow 150 dispersions with z-average hydrodynamic diameters of 67.04 and 69.30 nm, polydispersity indices of 0.12 and 0.15, zeta potentials near −40 mV, and low apparent viscosities. In contrast, BA-C produced a coarse dispersion with a z-average hydrodynamic diameter of 1858 nm, while BYK-190 gave 124.70 nm at the tested nominal dosage. The BA-A and BA-B solutions exhibited calculated Washburn penetrativities approximately 2.5 times that of water, and their pigment dispersions showed dominant proton transverse-relaxation peaks at shorter times than the BA-C dispersion. Overall, BA-A and BA-B exhibited more favorable powder wetting and colloidal stabilization than BA-C, supporting a formulation-specific relationship between molecular architecture and dispersion performance within the investigated series.

## 1. Introduction

Nanoscale pigment dispersions are widely used in inkjet printing [1,2], display color filters [3], and optical coatings [4,5] because they can provide high color strength, vividness, and transparency. When dispersed particle dimensions become sufficiently smaller than visible-light wavelengths, reduced light scattering can improve transparency outside the pigment absorption band [4]. Organic pigment primary particles are commonly several tens of nanometers in size, but their high specific surface area and strong interparticle attractions promote aggregation in liquid media [6]. Stable dispersion therefore requires efficient wetting of the powder, disruption of aggregates, and suppression of subsequent re-agglomeration [7,8].

Dispersants control this sequence through pigment-affine groups and solvated segments that provide electrostatic and/or steric stabilization [9]. Polymeric dispersants with acrylic, polyurethane, polyester, and related architectures have therefore been used for a wide range of organic pigments [9,10,11,12,13]. For example, Yang et al. [11] prepared a copolymeric dispersant containing styrene- and 2-(dimethylamino)ethyl methacrylate-based anchoring units for dispersing phthalocyanine blue, reporting a particle size of 340 nm for their optimized color paste. Zeng et al. [12] showed that the anchoring-group chemistry of polymeric dispersants affected the stability of Pigment Red 122. Although polymeric dispersants are versatile, structurally simpler pigment-derived molecules offer a complementary approach to combining pigment-related motifs and hydrophilic functionality within a compact molecular framework.

Pigment derivatives and structurally related synergists are attractive because their conjugated motifs can associate with organic pigment surfaces, while ionic substituents promote compatibility with water. Namgoong et al. [3] reported carboxyl-functional phthalocyanine derivatives for nanoparticle dispersion and color-filter applications. For Y150-related systems, Song et al. [14] prepared the benzoic-acid-functional synergist SY-43 and reported increased solubility relative to the parent pigment. Jeong et al. [15] introduced sulfonic-acid functionality into the Y150-related synergist PY-150ASA2 and observed improved aqueous compatibility and a smaller particle size than with the commercial synergist Solsperse 22000 under their test conditions. These studies suggest that pigment affinity and compatibility with the dispersion medium should be considered together when designing small-molecule dispersants [3,14,15]. Building on these functionalization strategies, a further question is how terminal functionality and conjugated linkage in compact barbiturate derivatives relate to both powder wetting and the resulting aqueous dispersion state.

In this study, BA-A, BA-B, and BA-C were selected as a comparative molecular set sharing a barbiturate-derived pigment-affine motif but differing in conjugated linkage and ionic terminal group. BA-A and BA-B compare different terminal functionalities within an arylazo/hydrazone-type framework, whereas BA-B and BA-C compare different conjugated linkages while retaining carboxyl functionality. The acid-form precursors were structurally characterized, and their potassium derivatives were evaluated as the sole added dispersants in terms of powder wettability, hydrodynamic size, zeta potential, viscosity, stability, morphology, optical transmission, and low-field-NMR proton relaxation behavior. BYK-190 was included as a commercial performance benchmark. The objective was to identify experimentally supported molecular–architecture–property correlations by relating structural differences to powder wetting and aqueous dispersion performance, thereby providing guidance for the selection of barbiturate-derived dispersants.

## 2. Materials and Methods

### 2.1. Materials

Barbituric acid (AR, 98%) was supplied by Beijing InnoChem Science & Technology Co., Ltd. (Beijing, China). Sulfanilic acid (AR, 99.8%), 4-aminobenzoic acid (AR, 99%), 4-formylbenzoic acid (AR, 98%), hydrochloric acid (AR, 36–38%), sodium nitrite (AR, 99%), sodium hydroxide (AR, 96%), and absolute ethanol (AR, 99.7%) were obtained from Sinopharm Chemical Reagent Co., Ltd. (Shanghai, China). Analytical-grade potassium hydroxide and n-hexane were used for salt formation and Washburn calibration, respectively. Pigment Yellow 150 (Y150) was obtained from LANXESS. The commercial aqueous dispersant BYK-190 was used as received. The manufacturer identifies it as an aqueous solution of a polyether-modified styrene-maleic anhydride copolymer with a typical non-volatile content of 40% [16]. Unless otherwise stated, all reagents were used without further purification.

### 2.2. Synthesis of Barbiturate Derivatives

The chemical structures of the potassium barbiturate derivatives BA-A, BA-B, and BA-C are shown in Figure 1. Their synthesis procedures are described below.

#### 2.2.1. Potassium (E)-4-[(2,4,6-Trioxohexahydropyrimidin-5-Yl)diazenyl]benzenesulfonate (BA-A)

Sulfanilic acid (0.01 mol) was dissolved in H_2_O (10 mL) containing concentrated HCl (0.01 mol), cooled to 5 °C, and treated with a cold aqueous solution of NaNO_2_ (0.01 mol). The resulting diazonium solution was added gradually to an ice-cold solution of barbituric acid (0.01 mol) in 10 mL of aqueous NaOH (0.02 mol), while the temperature was maintained below 5 °C. The reaction mixture was acidified to pH 6 with dilute HCl to precipitate the deep-yellow acid form, BA-a. The product was collected by filtration, washed with distilled water, and dried (yield: 85.68%). BA-a was neutralized with aqueous KOH at a 1:1 molar ratio to generate the potassium salt BA-A.

#### 2.2.2. Potassium (E)-4-[(2,4,6-Trioxohexahydropyrimidin-5-Yl)diazenyl]benzoate (BA-B)

4-Aminobenzoic acid (0.01 mol) was dissolved in H_2_O (10 mL) containing concentrated HCl (0.01 mol), cooled to 5 °C, and treated with a cold aqueous solution of NaNO_2_ (0.01 mol). The resulting diazonium solution was added gradually to an ice-cold solution of barbituric acid (0.01 mol) in 10 mL of aqueous NaOH (0.02 mol), while the temperature was maintained below 5 °C. The reaction mixture was acidified to pH 6 with dilute HCl to precipitate the deep-yellow acid form, BA-b. The product was collected by filtration, washed with distilled water, and dried (yield: 83.18%). BA-b was neutralized with aqueous KOH at a 1:1 molar ratio to generate the potassium salt BA-B.

#### 2.2.3. Potassium 4-[(2,4,6-Trioxotetrahydropyrimidin-5(2H)-ylidene)methyl]benzoate (BA-C)

4-Formylbenzoic acid (0.01 mol) was dissolved in ethanol (20 mL), and barbituric acid (0.01 mol) was dissolved separately in ethanol (30 mL). The solutions were combined and heated under reflux with stirring. A dark-red precipitate formed during the reaction. After 5 h, the hot mixture was filtered, and the precipitate was washed with ethanol and dried to obtain the acid form BA-c (yield: 87.57%). BA-c was treated with aqueous KOH at a 1:1.5 molar ratio to generate the water-soluble potassium salt BA-C. The precursor-to-KOH molar ratios reported above describe reagent inputs rather than experimentally determined degrees of neutralization.

### 2.3. Preparation of Pigment Dispersions

BA-A, BA-B, or BA-C was dissolved in deionized water in a jacketed beaker, followed by the addition of Y150. Unless otherwise specified for the optimization experiments, the pigment solids content was 5 wt% and the dispersant dosage was 7.5 wt% relative to the pigment mass. The mixture was processed at 5 °C for 30 min using an ultrasonic homogenizer (JY99-IIDN, Scientz, Ningbo, China) in pulse mode (2 s on/2 s off). The resulting dispersions were denoted Y150-BA-A, Y150-BA-B, and Y150-BA-C. A commercial control containing BYK-190 was prepared under the same nominal additive dosage and processing conditions and was denoted Y150-BYK-190. At a nominal dosage of 7.5 wt% relative to the pigment, the as-supplied BYK-190 contributes approximately 3.0 wt% non-volatile material based on the manufacturer’s typical value. The comparison is therefore on an as-supplied additive-mass basis, not an equivalent active-mass or molar basis; the BYK-190 dosage was not independently optimized.

### 2.4. Stability Testing

For the optimization experiments, z-average diameters were measured initially and after storage at 25 °C for 7 and 30 d. For the optimized formulations, the storage stability index (*S_ST_*) was evaluated after storage at 25 °C for 30 d according to Equation (1).

(1) $$S_{ST}=(1-\frac{D_{ST}-D_{0}}{D_{0}})\times 100\%$$

The thermal stability index (*S_T_*) was determined after heating the dispersions at 60 °C for 7 d [Equation (2)].

(2) $$S_{T}=(1-\frac{D_{T}-D_{0}}{D_{0}})\times 100\%$$

Retention after one freeze–thaw treatment (*S_F_*) was determined after freezing at −10 °C for 24 h and thawing at 60 °C for 24 h [Equation (3)].

(3) $$S_{F}=(1-\frac{D_{F}-D_{0}}{D_{0}})\times 100\%$$

Here, D_0_ is the initial z-average diameter, and D*_ST_*, D*_T_*, and D*_F_* are the values measured after storage, thermal treatment, and freeze–thaw treatment, respectively. All diameters were determined by dynamic light scattering (DLS) and are expressed in nm. An index of 100% denotes no change in z-average diameter; values below 100% indicate particle-size growth, whereas values slightly above 100% reflect a small decrease relative to the initial measurement. These indices describe changes in the measured z-average diameter and do not directly quantify sedimentation or sediment redispersibility.

### 2.5. Powder Wettability Evaluated by the Washburn Method

The Washburn capillary-rise method was used to determine the apparent contact angle of each liquid on the Y150 powder bed. A 0.5 g portion of Y150 powder was placed in a dedicated glass tube with a sintered-glass base covered by a single layer of filter paper. The tube was tapped against the benchtop ten times under its own weight for preliminary compaction. The powder bed was then compressed for 30 s using a cylindrical iron rod loaded with a 1 kg weight. The final bed height was kept consistent across samples. The relationship among mass uptake, liquid properties, the powder-bed constant, and contact angle is given by Equation (4) [17]:

(4) $$\cos\theta =\frac{\eta }{c\rho^{2}\gamma }\frac{d(m^{2})}{dt}$$

Here, *m* is the absorbed liquid mass (kg), t is time (s), η is dynamic viscosity (Pa·s), ρ is liquid density (kg·m^−3^), γ is surface tension (N·m^−1^), and c is the packed-bed capillary constant (m^5^). The apparent contact angle θ is reported in degrees, while cos θ is dimensionless. The value of c was calibrated using n-hexane, for which complete wetting (θ = 0°) was assumed. The slope d(m^2^)/dt (kg^2^·s^−1^) was used to determine the apparent contact angles of water and the dispersant solutions under the same powder-packing protocol. These are apparent powder-bed contact angles whose values depend on bed packing and calibration.

### 2.6. Characterization

The acid-form intermediates BA-a, BA-b, and BA-c were characterized by ^1^H NMR spectroscopy (600 MHz, Bruker, Karlsruhe, Germany; DMSO-d_6_), FTIR spectroscopy (Nicolet iS50, Thermo Fisher Scientific, Madison, WI, USA; 500–4000 cm^−1^), and UPLC-MS (Waters, Milford, MA, USA). Hydrodynamic size distributions were measured by DLS using a Nano-ZS90 analyzer (Malvern Instruments, Malvern, UK). Before DLS measurements, the dispersions were diluted with deionized water to a final pigment solids content of 0.01 wt% (corresponding to a 500-fold dilution for the standard 5 wt% dispersions). Measurements were performed at 25 °C after an equilibration time of 120 s using a scattering angle of 90°. The number and duration of sub-runs were selected automatically by the instrument software, and the z-average hydrodynamic diameter and polydispersity index (PDI) were obtained directly from the software. For each experimental condition and time point, particle size was measured three times on the same diluted dispersion sample, and the results are reported as mean ± standard deviation. The reported particle sizes are z-average hydrodynamic diameters rather than direct measurements of primary pigment particle size. Zeta potentials were measured using the same instrument in water at 25 °C. Particle morphology was examined by cold-field-emission scanning electron microscopy (Regulus 8100, Hitachi, Tokyo, Japan). Powder contact angles and surface tensions were measured with a DCAT25 tensiometer (DataPhysics, Filderstadt, Germany), with surface tension determined at 20 °C using the platinum-plate method. Apparent viscosity was measured using a HAAKE MARS 60 rotational rheometer (Thermo Scientific, Karlsruhe, Germany) over a shear-rate range of 10–500 s^−1^, with 50 measurement points collected over 300 s. Optical transmittance spectra were obtained using a 1 mm quartz cuvette, whereas CIE 1931 chromaticity measurements used a 10 mm double-sided transmission quartz cuvette. Proton transverse-relaxation distributions were acquired by low-field NMR (PQ001, Niumag, Shanghai, China). The T_2_ inversion range was 0.01–10,000 ms, using 100 logarithmically spaced T_2_ grid points. The resulting T_2_ distribution amplitude was used for the relaxation-distribution plots. For particle size results, error bars represent standard deviations of these three repeated measurements (n = 3).

## 3. Results

### 3.1. Molecular Structures and Spectroscopic Characterization

The ^1^H NMR spectra of BA-a, BA-b, and BA-c are shown in Figure 2(A_1_–A_3_). The arylazo derivatives BA-a and BA-b can undergo azo-hydrazone tautomerism, and their spectra in DMSO-d_6_ are consistent with hydrazone-dominant forms, although their tautomer populations were not quantified. For BA-a, the signals at δ 6.65–6.83 ppm were assigned to aromatic protons, while those at δ 10.45–10.63 ppm were assigned to the barbituric imide N-H protons. The markedly downfield signal at δ 13.29 ppm was assigned to an exchangeable hydrazone N-H environment rather than to the sulfonic acid proton, further supporting this interpretation. BA-b and BA-c exhibited aromatic signals at δ 7.47–7.92 and 7.99–8.04 ppm, respectively. For BA-c, the signal at δ 8.30 ppm was assigned to the arylidene proton, while that at δ 13.19 ppm was consistent with the carboxylic acid proton.

UPLC-MS gave major ions at *m*/*z* 313, 275, and 261 for BA-a, BA-b, and BA-c, respectively, consistent with their expected molecular masses. Together with the ^1^H NMR and FTIR results, these data support formation of the three acid-form precursors. The water-soluble potassium salts BA-A, BA-B, and BA-C used in the dispersion experiments were subsequently generated by the KOH neutralization procedures described above. The purity and degree of neutralization of the final potassium derivatives were not independently quantified.

The three dispersants therefore represent two molecular-linkage classes: arylazo/hydrazone-type derivatives (BA-A and BA-B) and an arylidene derivative (BA-C), together with sulfonate or carboxylate ionic termini. The present spectra establish molecular connectivity and functional-group differences but do not uniquely determine solution-phase tautomer populations, E/Z configuration, or the dihedral angle between the aromatic and barbiturate units. Accordingly, subsequent discussion uses the term molecular architecture and does not attribute dispersion performance solely to assumed planarity.

### 3.2. Molecular-Architecture-Dependent Dispersion Behavior

Figure 3 compares the effects of dispersant dosage, pigment solids content, and ultrasonication time on initial and 7 d z-average diameter. Increasing the BA-A dosage from 1 to 7.5 wt% relative to pigment reduced the initial diameter from 82.70 nm to the mid-60 nm range, whereas increasing the dosage to 10 wt% produced little additional benefit (Figure 3(A_1_)). This performance plateau identifies 7.5 wt% as a practical dosage but does not, without adsorption measurements, demonstrate surface saturation. Increasing the pigment solids content above 20 wt% increased particle size and reduced 7 d retention; 5 wt% was therefore used for the subsequent comparison. Particle size decreased with ultrasonication time, but longer treatment did not improve 7 d stability, so 30 min was selected. BA-B showed trends comparable to BA-A. BA-C remained in the micrometer range under all screened conditions, indicating that its inferior performance could not be overcome simply by changing dosage, solids content, or processing time. The molecular origin of this difference cannot be assigned solely from the present data.

Figure 4 shows dry-state morphologies of pristine and dispersed Y150. The untreated pigment formed irregular micrometer-scale aggregates with poorly resolved particle boundaries (Figure 4(A_1_,A_2_)). Samples prepared with BA-A and BA-B contained more clearly separated nanoscale particles and substantially fewer large aggregates (Figure 4(B_1_,B_2_,C_1_,C_2_)). BA-C also promoted partial deagglomeration during processing, but extensive secondary aggregation remained after drying (Figure 4(D_1_,D_2_)). Because FE-SEM examines the dried state, these images are interpreted qualitatively and are not used as a direct measurement of the DLS hydrodynamic diameter.

Table 1 summarizes the properties of the Y150 dispersions. BA-A and BA-B produced z-average hydrodynamic diameters of 67.04 and 69.30 nm, respectively, with narrow size distributions (PDI 0.12 and 0.15), large negative zeta-potential magnitudes near −40 mV, and low apparent viscosities of 1.16 and 1.38 mPa·s. The numerical difference between their mean diameters is reported descriptively and is not interpreted as evidence that either dispersant provides superior particle-size reduction. Their storage and thermal stability indices remained close to 100%. BA-C, by contrast, was already strongly aggregated before accelerated treatment (1858 nm; PDI 0.42), weakly charged (−9.78 mV), and much more viscous (16.72 mPa·s). Its storage index near 100% therefore does not indicate a useful nanoscale dispersion; the coarse initial state must be considered together with the index. At the tested dosage, BYK-190 gave an intermediate diameter of 124.70 nm and a zeta potential of −38.18 mV. BA-A and BA-B showed higher storage and thermal stability indices than BYK-190, whereas BYK-190 exhibited the highest freeze–thaw retention index (92.38%, compared with 79.93% and 80.38% for BA-A and BA-B, respectively). These results indicate complementary rather than uniformly superior performance at the tested nominal dosages. The low viscosity and large negative zeta-potential magnitude of the BA-A and BA-B systems are consistent with reduced flocculation and effective electrostatic stabilization. In contrast, the aggregated BA-C system combined a small zeta-potential magnitude with high viscosity. These correlations do not by themselves quantify adsorption strength or surface coverage.

Figure 5 compares the visual appearance, particle-size distributions, and optical behavior of the four dispersions. The background lettering remained readily visible through Y150-BA-A and Y150-BA-B, whereas Y150-BA-C appeared visibly turbid (Figure 5A). The distributions in Figure 5B agree with the z-average trends in Table 1: BA-A and BA-B show narrow nanoscale populations, BYK-190 is broader and centered at a larger diameter, and BA-C is centered in the micrometer range. The transmittance spectra in Figure 5C show the intended long-wavelength transparency comparison, while the CIE 1931 coordinates in Figure 5D place all four samples in the yellow-orange region. BA-A and BA-B nearly overlap in chromaticity, indicating that their similar dispersion states preserve comparable hue.

### 3.3. Molecular Structure, Wetting, and Interfacial Proton Environment

Table 2 compares the liquid properties and apparent contact angles that govern capillary wetting of the Y150 powder bed. In the Lucas–Washburn regime, the instantaneous penetration velocity in an ideal cylindrical capillary is given by Equation (5) [17]:

(5) $$v=\frac{dl}{dt}=\frac{r\gamma cos\theta }{4\eta l}=\frac{rP}{2l}$$

Here, *v* is the instantaneous penetration velocity (m·s^−1^), and *r* and *l* are the effective capillary radius and penetration length (m), respectively; *γ*, *θ*, and *η* have the meanings and units defined after Equation (4). Defining the Washburn penetrativity as *P* = *γ cos θ*/(2*η*) gives *v* = *rP*/(2*l*). P has units of m·s^−1^ and is not a dimensionless normalized coefficient or a directly measured powder-bed penetration velocity. The P values for BA-A, BA-B, BA-C, BYK-190, and water were 41.28, 41.13, 14.22, 3.08, and 16.32 m·s^−1^, respectively. The dimensionless ratios P/Pwater are reported separately in Table 2; BA-A and BA-B give ratios of approximately 2.5. The numerical difference between BA-A and BA-B is not interpreted as statistically resolved. The low *P* value of BYK-190, despite its ability to produce a 124.70 nm dispersion, also shows that the calculated wetting parameter alone does not determine final dispersion performance.

Figure 6A,B compare the proton transverse-relaxation distributions of the three Y150 dispersions. For descriptive purposes, the spectra are divided into short- (0.01–10 ms), intermediate- (10–1000 ms), and long-T_2_ (1000–10,000 ms) windows. These operational windows are not treated as unique assignments to specific water species. Y150-BA-A and Y150-BA-B show dominant peaks at shorter relaxation times than Y150-BA-C. The relaxation response is sensitive to accessible particle surfaces and solvent–surface interactions [18]. However, the dispersions differ markedly in hydrodynamic particle size and may also differ in accessible interfacial area. Proton exchange and solution composition may additionally influence the relaxation response. The observed relaxation differences should consequently be interpreted as changes in the overall aqueous/interfacial state rather than as direct measurements of dispersant adsorption, surface coverage, or a specific molecular orientation. The LF-NMR results therefore provide complementary evidence of different aqueous and interfacial proton environments in the three dispersions.

The combined results suggest a working hypothesis in which powder wetting and interfacial association contribute to the stabilization of Y150 dispersions. Improved capillary wetting may facilitate liquid penetration into pigment agglomerates and displacement of trapped air, assisting agglomerate breakup during ultrasonication. The conjugated barbiturate-derived moieties may associate with pigment surfaces, while the solvated ionic termini may contribute to electrostatic repulsion and suppress subsequent re-agglomeration. Compared with BA-C, the BA-A and BA-B solutions exhibit higher calculated Washburn penetrativities, and their pigment dispersions show more negative zeta potentials and lower apparent viscosities. This combination is consistent with more favorable powder wetting and reduced particle association. The dominant proton transverse-relaxation peaks of the BA-A and BA-B dispersions also occur at shorter times than the corresponding peak of the BA-C dispersion, providing complementary information on differences in the aqueous and interfacial proton environment.

## 4. Conclusions

A comparative molecular–architecture–property relationship was established for three barbiturate-derived dispersants. The arylazo/hydrazone-type BA-A and BA-B produced Y150 dispersions with z-average diameters of 67.04 and 69.30 nm, PDIs of 0.12 and 0.15, and zeta potentials of −40.03 and −40.13 mV, respectively, as well as low apparent viscosities. In contrast, the arylidene-type BA-C produced a strongly agglomerated dispersion with a z-average diameter of 1858 nm, a PDI of 0.42, and a zeta potential of −9.78 mV. At the tested nominal dosage, BYK-190 produced a z-average diameter of 124.70 nm with a PDI of 0.17 and a zeta potential of −38.18 mV. BA-A and BA-B also exhibited calculated Washburn penetrativities approximately 2.5 times that of water, together with dominant LF-NMR proton-relaxation peaks at shorter times than BA-C. Overall, BA-A and BA-B showed more effective pigment-powder wetting and aqueous colloidal stabilization than BA-C, establishing a clear molecular–architecture–property relationship within the investigated series.

## Figures and Tables

**Figure 1:**
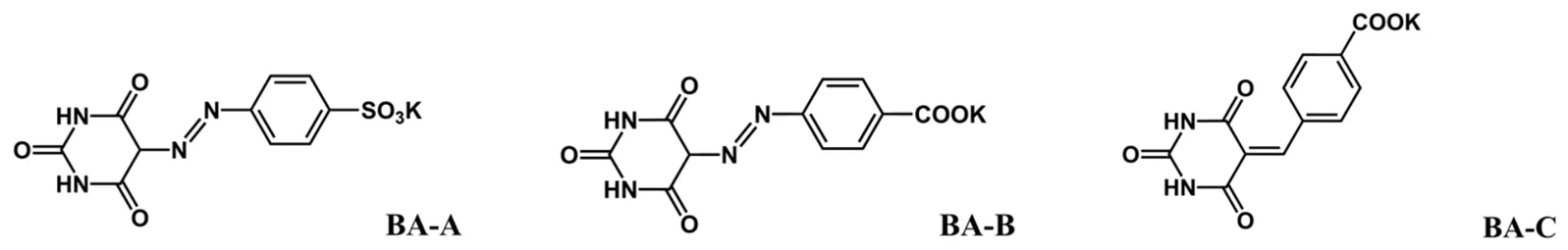
Chemical structures of the potassium barbiturate derivatives BA-A, BA-B, and BA-C.

**Figure 2:**
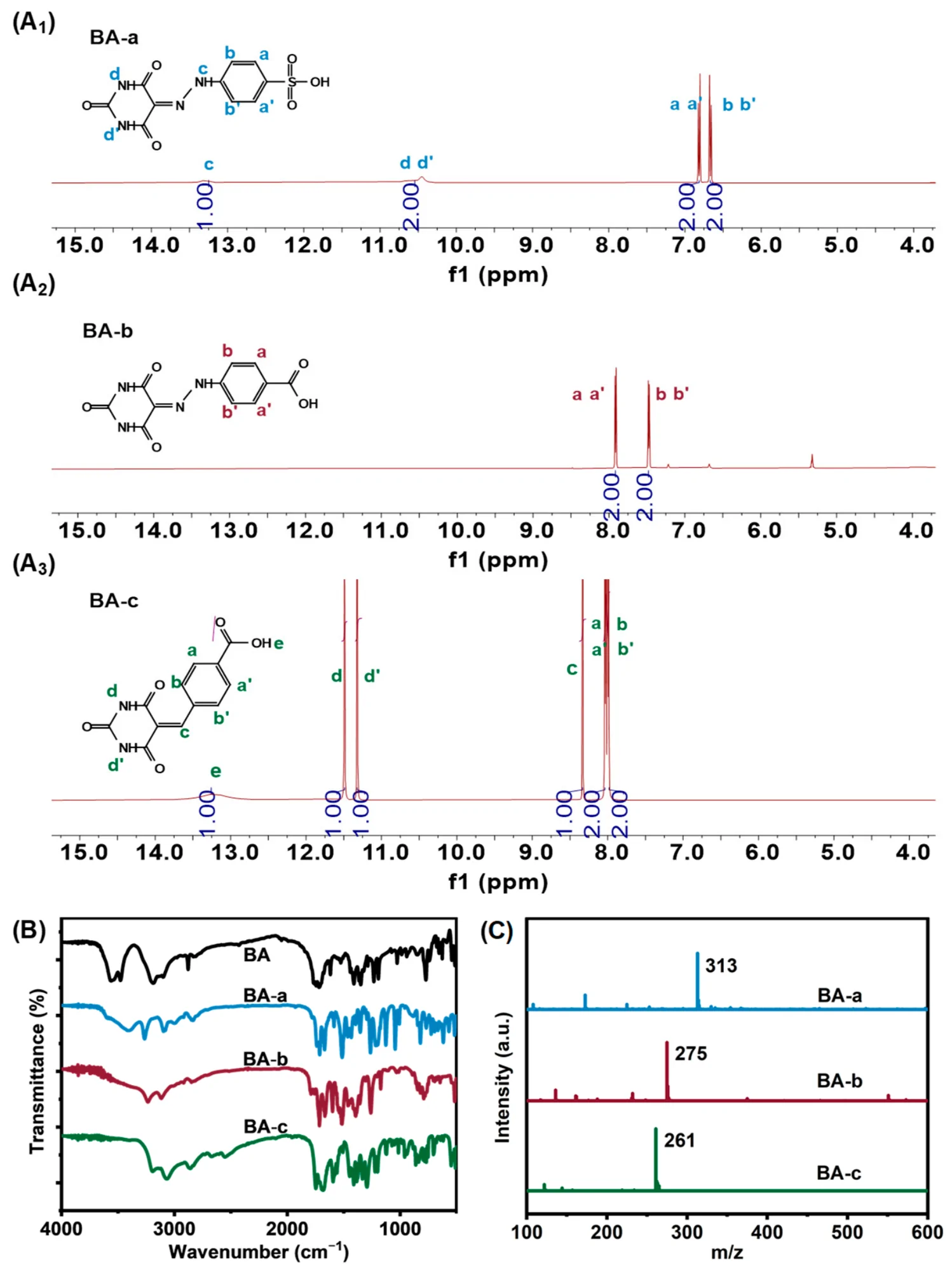
Structural characterization of the acid-form precursors BA-a, BA-b, and BA-c: (**A_1_**–**A_3_**) ^1^H NMR spectra, (**B**) FTIR spectra, and (**C**) UPLC-MS spectra.

**Figure 3:**
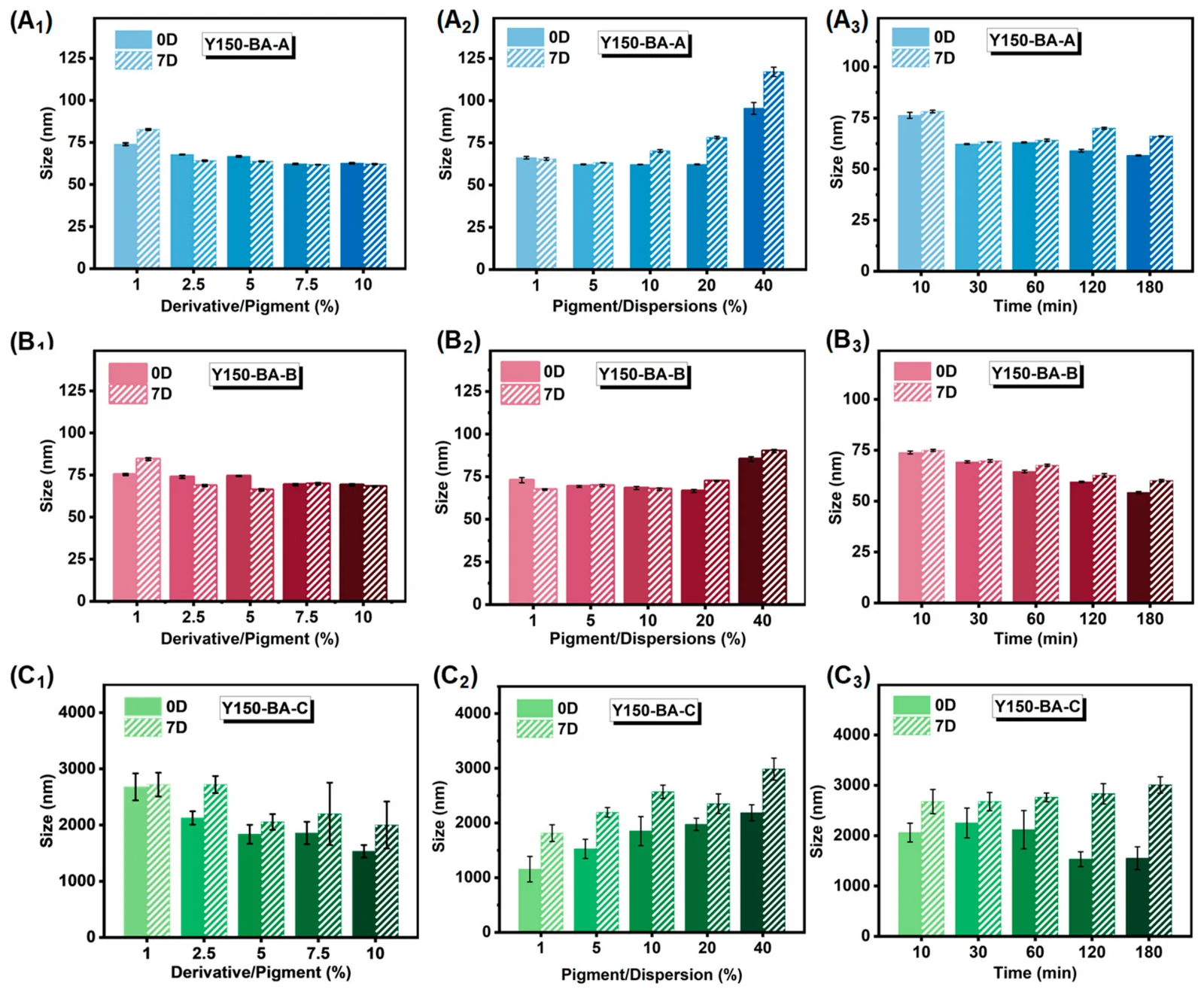
Effects of (**A_1_**,**B_1_**,**C_1_**) dispersant-to-pigment mass ratio, (**A_2_**,**B_2_**,**C_2_**) Y150 solids content, and (**A_3_**,**B_3_**,**C_3_**) ultrasonication time on the initial (0 d) and 7 d z-average diameters of dispersions prepared with (**A**) BA-A, (**B**) BA-B, and (**C**) BA-C. Data represent the means of three repeated measurements of the same dispersion sample for each experimental condition and time point (technical replicates, n = 3); error bars indicate standard deviations.

**Figure 4:**
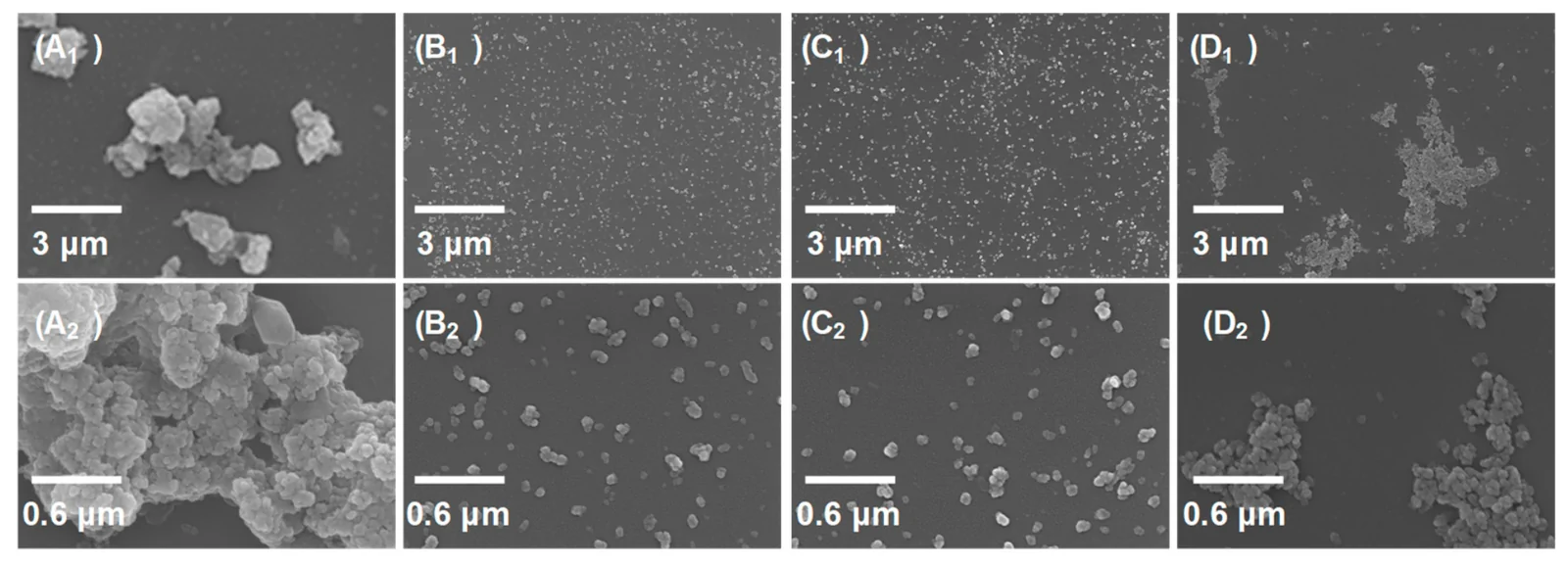
FE-SEM images of (**A_1_**,**A_2_**) pristine Y150 and Y150 after dispersion with (**B_1_**,**B_2_**) BA-A, (**C_1_**,**C_2_**) BA-B, and (**D_1_**,**D_2_**) BA-C. Scale bars are 3 μm in the upper row and 0.6 μm in the lower row.

**Figure 5:**
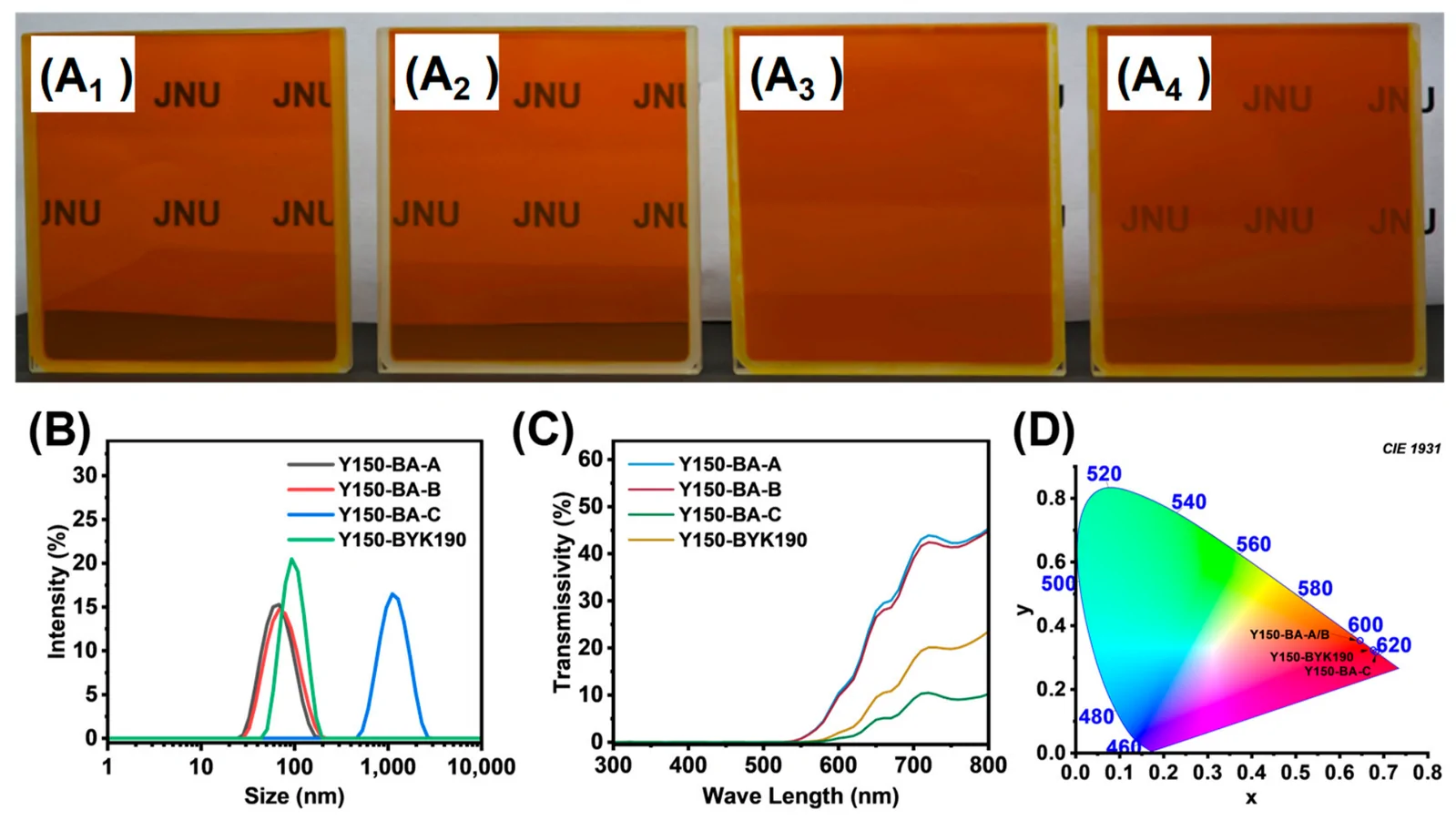
Photographs and particle-size/optical properties of Y150 dispersions: (**A_1_**–**A_4_**) Y150-BA-A, Y150-BA-B, Y150-BA-C, and Y150-BYK-190, respectively; (**B**) intensity-weighted particle-size distributions; (**C**) transmittance spectra; and (**D**) CIE 1931 chromaticity coordinates.

**Figure 6:**
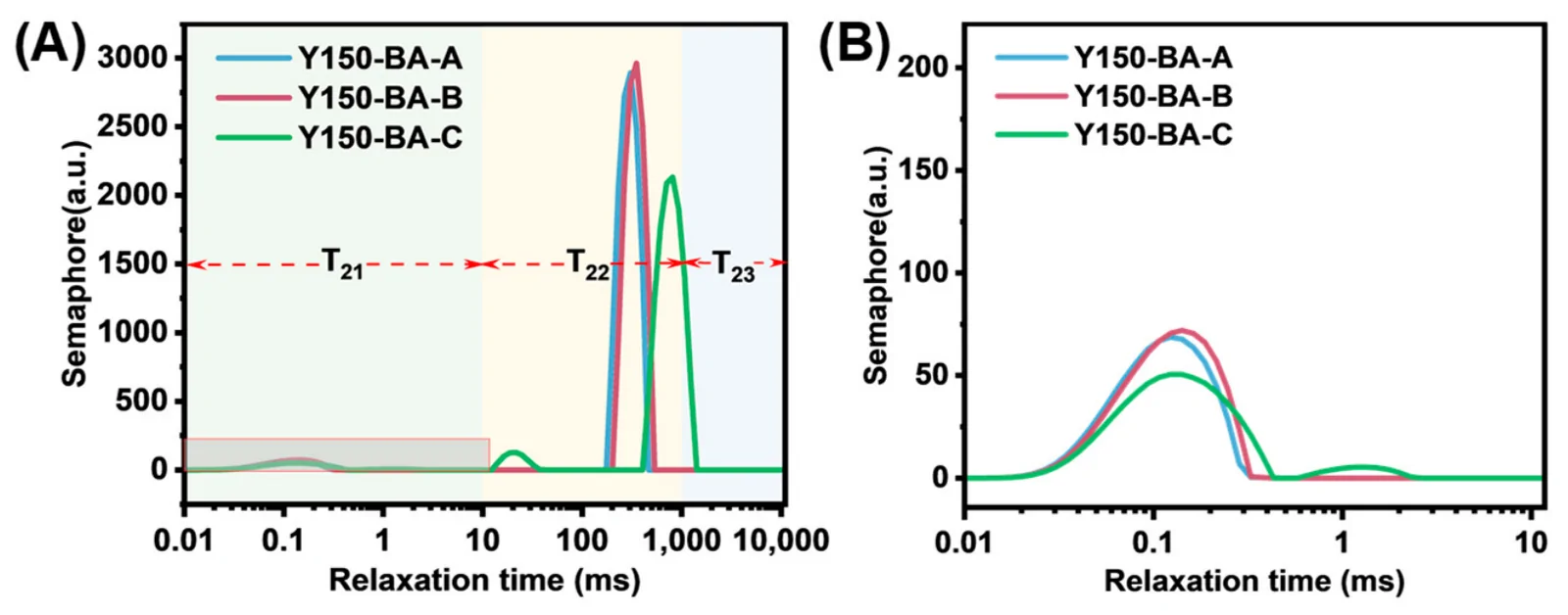
Low-field NMR proton transverse-relaxation distributions of Y150 dispersions: (**A**) full T_2_ distributions and (**B**) enlarged short-T_2_ region.

**Table 1:** Properties and particle-size stability indices of the optimized Y150 dispersions.

Property	Y150-BA-A	Y150-BA-B	Y150-BA-C	Y150-BYK-190
z-average diameter (nm)	67.04	69.30	1858.00	124.70
PDI	0.12	0.15	0.42	0.17
Zeta potential (mV)	−40.03	−40.13	−9.78	−38.18
Apparent viscosity (mPa·s)	1.16	1.38	16.72	2.04
Storage stability index, S_ST_ (%)	100.10	98.95	102.48	87.92
Thermal stability index, S_T_ (%)	98.37	95.82	50.25	57.28
Freeze–thaw retention index, S_F_ (%)	79.93	80.38	62.75	92.38

**Table 2:** Wetting parameters of the dispersant solutions on Y150 powder.

Property	Water	BA-A Solution	BA-B Solution	BA-C Solution	BYK-190 Solution
Surface tension (mN·m^−1^)	72.03	69.82	70.72	57.60	42.37
Apparent powder-bed contact angle (°)	66.22	11.03	15.13	62.99	72.77
Dynamic viscosity (mPa·s)	0.89	0.83	0.83	0.92	2.04
Washburn penetrativity, P (m·s^−1^)	16.32	41.28	41.13	14.22	3.08
Dimensionless ratio, P/P_water_	1.00	2.53	2.52	0.87	0.19

## Data Availability

The original contributions presented in this study are included in the article. Further inquiries can be directed to the corresponding author.
